# Drug Delivery Systems for the Oral Administration of Antimicrobial Peptides: Promising Tools to Treat Infectious Diseases

**DOI:** 10.3389/fmedt.2021.778645

**Published:** 2022-01-25

**Authors:** Caroline Deshayes, Md. Nasir Arafath, Véronique Apaire-Marchais, Emilie Roger

**Affiliations:** ^1^University of Angers, INRAE, SIFCIR, SFR QUASAV, Angers, France; ^2^University of Angers, INSERM, CNRS, MINT, SFR ICAT, Angers, France

**Keywords:** oral route, antimicrobial peptides (AMPs), infectiology, pharmaceutical forms, drug delivery systems (DDS)

## Abstract

Antimicrobial peptides (AMPs) have a great potential to face the global expansion of antimicrobial resistance (AMR) associated to the development of multidrug-resistant (MDR) pathogens. AMPs are usually composed of 10–50 amino acids with a broad structural diversity and present a range of antimicrobial activities. Unfortunately, even if the oral route is the most convenient one, currently approved therapeutic AMPs are mostly administrated by the intravenous route. Thus, the development of novel drug delivery systems (DDSs) represents a promising opportunity to protect AMPs from chemical and enzymatic degradation through the gastrointestinal tract and to increase intestinal permeability leading to high bioavailability. In this review, the classification and properties as well as mechanisms of the AMPs used in infectiology are first described. Then, the different pharmaceutical forms existing in the market for oral administration are presented. Finally, the formulation technologies, including microparticle- and nanoparticle-based DDSs, used to improve the oral bioavailability of AMPs are reviewed.

## Introduction

Almost 100 years after the discovery of antibiotics, an increasing number of multidrug-resistant (MDR) pathogens are alarming. The overuse and misuse of antibiotics have led to antimicrobial resistance (AMR), which is one of the most important public health threats worldwide (1–4). AMR occurs when bacteria, viruses, or fungi develop resistance to antibiotics, antiviral, or antifungal drugs, respectively (5, 6). At least 700,000 people die annually worldwide due to drug-resistant diseases. Worst-case projections estimate that AMR could cause 10 million deaths each year by 2050, leading to a global economic output of 100 trillion USD (7). AMR is a complex issue of global concern with potentially dramatic health and economic consequences. In 2015, WHO launched the Global Action Plan on AMR and the Global AMR and Use Surveillance System (GLASS) (8, 9). In this context, antimicrobial peptides (AMPs) provide a great potential as an alternative strategy to fight against MDR microorganisms (10–12). AMPs are synthetic or naturally conserved molecules found in organisms ranging from prokaryotes to humans and are part of the body's first line of defense against pathogens (bacteria, viruses, fungi, and parasites). Most of them are positively charged with hydrophobic residues with a broad spectrum of antimicrobial activities. For decades, AMPs have shown a growing interest as potential therapeutic agents. Several hundreds of AMPs are under preclinical and clinical development, nevertheless, only a few AMPs are currently approved (13). They are used for the treatment of infectious diseases like bacterial skin infections, *Clostridium difficile* pseudomembranous colitis, HIV infection, or *Candida* infections. Thus, AMPs are a promising class of molecules active against bacteria (14), viruses (15), or fungi (16). Furthermore, they could be effective against MDR pathogens and have a potent activity against intracellular bacteria and also against the biofilms that are involved in antibiotic resistance (12, 17–20).

Now, for several reasons, the AMPs that are used as therapeutic agents are mainly administrated by the intravenous route rather than by the oral route. Indeed, the gastrointestinal environment affects the drug stability, solubility, and permeability across the mucosal barriers. In addition, due to their nature, AMPs have a low bioavailability and solubility, are easily degraded by proteases, and potentially present toxicity and immunogenicity, which limit their use as antimicrobial agents. However, the oral route has several advantages: easy to use, non-invasiveness, and convenience for self-administration (21). To minimize the drawbacks of AMPs and the oral route, the development of new drug delivery systems (DDSs) is required (10, 22, 23). This review describes (i) the properties and activities of the AMPs that are used against microbial pathogens, (ii) their indications for oral administration, and (iii) the micro- and nano-DDSs to improve oral bioavailability for local and systemic drug delivery.

## AMPs: Classification and Properties

The history of AMPs began in the early 1920s with the discovery by Alexander Fleming of a bacteriolytic substance in the tissues and secretions of human and animals, and in some vegetable tissues (24, 25). The name “lysozyme” has been applied to this enzyme, and the peptidic nature of this salivary antiseptic was subsequently established. Since this discovery nearly a century ago, the interest in AMPs has continued to grow, as evidenced by the number of articles published on this topic with an average of 15,000 articles per year in the last decade. Due to the pleiotropic functions not only killing microbes but also controlling host physiological functions such as inflammation, angiogenesis, and wound healing, alternative terms for AMPs have also appeared like “host defense peptides,” “alarmins,” and even “defensins.” AMPs are synthesized by virtually all living organisms, from bacteria to humans *via* plants. AMPs represent the first line of defense against invading pathogens, being a key part of the innate immune system. AMPs are either ribosomally synthesized oligopeptides or non-ribosomally synthesized peptides. In the latter case, peptides are assembled by multimodular enzymes designated as non-ribosomal peptide synthetases (NRPS). Several regularly updated databases such as APD3 (26), the Collection of AMPs (CAMP) (27), the Database of Antimicrobial Activity and Structure of Peptides (DBAASP) (28), or the Data Repository of AMPs (DRAMP) (29) provide information on sequences, structures, activities, or clinical status of thousands of AMPs identified so far.

Antimicrobial peptides are usually 10–50 amino acids long and lower than 10 kDa, and they contain a composition rich in cationic and hydrophobic amino acids. However, AMPs lack any consensus amino acid sequence and present a broad structural variety and range of antimicrobial activities. The diversity of AMPs causes difficulty in their classification. AMPs can be classified in numerous ways: biological sources, peptide properties, structure, or activity.

### Classification Based on Biological Sources

**Figure 1:**
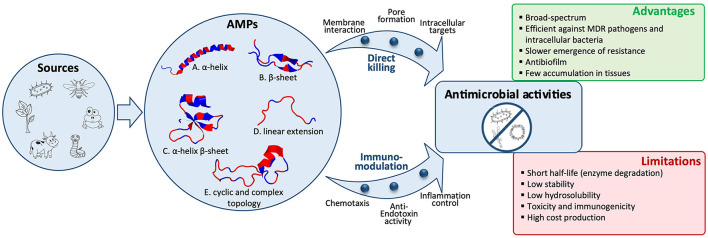
Structural diversity of antimicrobial peptides (AMPs) and their activities against bacteria, viruses, or fungi. A wide variety of biological sources, including microbes, insects, amphibians, reptiles, mammals, or plants, produce AMPs, which are classified into five structural classes. Representative examples of these five classes are shown as a cartoon representation and colored by hydrophobicity [sourced from the RCSB Protein Data Bank (https://www.rcsb.org/)]: (A) α-helical structure of human LL-37 (PDB entry: 2K6O); (B) β-sheet structure of bovine lactoferricin (PDB entry: 1LFC); (C) α-helix and β-sheet structure of human beta-defensin-1 (PDB entry: 1IJV); (D) Linear extension structure of bovine indolicidin (PDB entry: 1G89); (E) Cyclic structure of *Bacillus subtilis* Subtilisin A (PDB entry: 1PXQ). Direct pathogen killing and immunomodulatory activities of AMPs lead to antibacterial, antiviral, and antifungal activities. AMPs' advantages and limitations to treat infectious diseases are listed.

The biological sources of AMPs are wide. Indeed, natural peptides have been identified in all kingdoms of life, from bacteria, fungi, plants to animals (30, 31). Animal AMPs can be further classified into insect AMPs, amphibian AMPs, fish AMPs, reptile AMPs, and mammal AMPs (32–34) (Figure 1).

### Classification Based on Peptide Properties

Antimicrobial peptides can be classified based on peptide properties such as charge, amino acid composition, hydrophobicity, and length. AMPs are oligopeptides containing a varying number of amino acids (usually 10–50 amino acids, ranging in size from 2 to 10 kDa). They can be sorted by size: short (10–24 aa), medium (25–50 aa), and long (50–100 aa). Based on net charge, there can be cationic, neutral, and anionic peptides although most of them are cationic and display a net positive charge ranging from +2 to +13 and may contain a specific cationic domain (35). The cationic nature can be attributed to the presence of lysine and arginine (and sometimes histidine) residues, which allow them to interact with negatively charged bacterial cell membranes, causing the direct destabilization of the surface of membranes with pore formation and subsequent cell lysis. Based on amino acid composition, AMPs can be predominantly rich in specific amino acids such as proline (e.g., apidaecin and pyrrhocoricin), tryptophan, arginine, glycine, histidine (e.g., Histatin-5), or rare modified amino acids (e.g., nisin) (30, 36). Based on hydrophobicity, hydrophobic, amphipathic, and hydrophilic peptides exist. Hydrophobicity governs the extent to which AMPs will be able to be partitioned into the membrane lipid bilayer. It is required for membrane permeabilization, whereas amphipathicity determines whether AMPs can be inserted into the bacterial cell membranes to form hydrophobic channels or pores.

### Classification Based on Structure

Antimicrobial peptides are classically divided into four categories based on their structures, including linear α-helical peptides, β-sheet peptides, both α-helix and β-sheet peptides, and a linear extension structure. In addition to these four categories, a fifth referred to as the topologically complex AMPs, including various post-translational modifications (PTMs), has recently been added (37) (Figure 1).

The AMPs that adopt a linear α-helix structure represent the largest and best-studied group. They are predominantly found in the extracellular matrix of insects and amphibians like *magainin* from *Xenopus laevis* although some other well-known examples are produced by mammals like the human peptide *LL-37*, a member of cathelicidins (35, 37). Some of these AMPs are unstructured in solution and undergo conformational changes upon interactions with target membranes. Amidation at the C-terminus has been shown to increase its antimicrobial activity by stabilizing α-helical conformation and by eliminating the negative charge of the carboxyl group, therefore enhancing peptide binding to negatively charged target membranes (38). AMPs in the β-family are characterized by at least a pair of two β-strands in the structure. Almost all of these AMPs contain cysteine residues forming one or more disulfide bonds, which stabilize the structure (39). These peptides are therefore more structured in solution and do not undergo major structural changes in a membrane environment. The β-sheet peptides include, for example, *bovine lactoferricin* or *human defensins*. Some AMPs adopt a structure with both α-helix and β-sheet elements, such as the *cis* defensins superfamily (40). The fourth category represents AMPs with a linear extension structure, which do not fold into a particular 3D structure. They often contain a high proportion of certain amino acids such as arginine, tryptophan, or proline like *indolicidin* (37). A recent fifth group of AMPs has been proposed to gather AMPs with cyclic and complex topologies (37). These peptides do not adopt a linear structure unlike AMPs belonging to the first four classes. In this group, two types of cyclic AMPs are found: “head to tail” and “head to side chain” cyclic topologies. To stabilize their structure, most of the cyclic AMPs contain disulfide bonds or thioether bridges. Plant cyclotides represent a family of backbone-cyclic AMPs with three stabilizing disulfide bonds. Lasso peptides like bacterial *microcin* are part of head to side chain AMPs and consist of a macrolactam ring formed between the N-terminal α-amino group and an aspartate or glutamate side chain and a linear C-terminal peptide tail (41).

## AMPs for the Treatment of Infectious Diseases

Antimicrobial peptides exhibit several mechanisms of action for an interaction with bacteria and other microorganisms such as viruses and fungi. In general, AMPs kill microorganisms by disturbing membrane integrity or by interacting with the synthesis of intracellular components such as DNA, RNA, and proteins. They can also exert a broad range of immunomodulatory activities (Figure 1).

### Mechanisms of Action of AMPs

#### Antibacterial Activities

Membrane interaction is a key factor for the direct activity of AMPs. Many AMPs cause disruption of the physical activity of the microbial membrane and/or translocation across the membrane into the cytoplasm of bacteria to act on an intracellular target. Some of them are presented in Table 1. AMPs kill bacteria by disturbing membrane integrity through membrane lysis (i.e., *colistin, bacitracin, daptomycin*, and *polymyxin B*), membrane poration (i.e., *gramicidin D* and *tyrothricin*), or the inhibition of cell wall synthesis (i.e., *teicoplanin* and *vancomycin*) (Table 1). Gram-negative and Gram-positive bacteria have molecules at the surface that confer a negative charge, allowing an electrostatic interaction with cationic peptides. Thus, teichoic acids in the cell wall of Gram-positive bacteria and lipopolysaccharides in the outer membrane of Gram-negative bacteria provide additional electronegative charge to the bacterial surface. Several models have been proposed to explain the interaction of AMPs with the bacterial membrane. Thus, **three** possible pathways to disrupt the inner membrane have been described: (i) in the barrel-stave model, peptides can perpendicularly insert into the membrane, promoting peptide–peptide interactions thanks to the AMP's amphipathic structure and resulting in the formation of a peptide-lined transmembrane pore, (ii) concerning the toroidal-pore model, the insertion of the peptides is induced by a curvature in the lipid layer, and a pore is generated by both the peptide and the phospholipid head group, and (iii) in the carpet-model, the peptide is absorbed onto the membrane, covering the entire surface leading to the loss of the membrane integrity and the formation of micelles (Figure 1). Moreover, peptides can translocate into the cytoplasm and directly inhibit the cell wall and protein synthesis, bacterial cell division, or DNA replication by interacting with specific proteins involved in the biological process (18, 19, 48, 50).

**Table 1:** The antimicrobial peptide (AMP) drugs approved by the Food and Drug Administration (FDA).

**Antimicrobial peptide**	**Drug name**	**Source**	**Peptide type**	**Mechanism of action**	**Administration**	**Antimicrobial activity**	**Application**	**FDA approval**	**References**
**Antibacterial drug**									
Colistin (polymyxin E)	Coly-Micins^®^ Coli Genta AP-HP^®^, Koolistin^®^	*Bacillus colistinus*	Cyclic lipopeptide	Membrane lysis	Intravenous Oral (suspension)	Gram-negative bacteria	Bacterial infections	1962	(32, 42, 43)
Bacitracin	Baciim^®^	*Bacillus subtilis*	Cyclic polypeptide	Membrane lysis	Topical	Gram-positive bacteria	Skin and eye infections	1984	(13, 19, 42)
Dalbavancin	Dalvance^®^ Xydalba^®^	Semisynthetic derivative of teicoplanin	Lipoglycopeptide	Inhibitor of cell wall synthesis	Intravenous	Gram-positive bacteria	Acute bacterial skin infections	2014	(13, 19, 42)
Daptomycin	Cubicin^®^	*Streptomyces roseosporus*	Cyclic lipopeptide	Membrane lysis	Intravenous	Gram-positive bacteria	Bacterial skin infections	2003	(13, 19, 42)
Gramicidin D	Neosporin^®^	*Bacillus brevis*	Linear peptide	Membrane poration	Topical	Gram-positive bacteria	Bacterial conjunctivitis	1995	(42)
Oritavancin	Orbactiv^®^	Semisynthetic derivative of vancomycin	Lipoglycopeptide	Membrane lysis Inhibitor of cell wall synthesis	Intravenous	Gram-positive bacteria	Acute bacterial skin infections	2014	(13, 19, 42)
Polymyxin B	Poly-Rx^®^	*Bacillius polymyxa*	Cyclic lipopeptide	Membrane lysis	Intravenous	Gram-negative bacteria	Bacterial infections	1964	(42)
Teicoplanin	Targocid^®^ Teicomid^®^	*Actinoplanes teichomyceticus*	Lipoglycopeptide	Inhibitor of cell wall synthesis	Intravenous Oral	Gram-positive bacteria	Bacterial infections *Clostridium difficile* associated diarrhea	1990	(13, 19, 44)
Tyrothricin	Tyrozet^®^ Lemocin^®^ Dorothricin^®^ Anginovag^®^	*Bacillus brevis*	Linear peptide	Membrane poration	Oral (lozenge)	Gram-positive bacteria	Acute pharyngitis	N.D	(45, 46)
Telavancin	Vibativ^®^	Semisynthetic derivative of vancomycin	Lipoglycopeptide	Membrane lysis Inhibitor of cell wall synthesis	Intravenous	Gram-positive bacteria	Acute bacterial skin infections	2009	(13, 19, 42)
Vancomycin	Vancocin^®^ Vancomycin^®^ EG	*Streptomyces orientalis*	Lipoglycopeptide	Inhibitor of cell wall synthesis	Intravenous Oral (capsule or powder)	Gram-positive bacteria	Bacterial infections *Clostridium difficile* associated diarrhea	1983	(13, 19, 42, 47)
**Antiviral drug**									
Azatanavir	Reyataz^®^	Synthetic	Azapeptide protease inhibitor	Protease inhibitor	Oral (capsule)	Human immunodefiency virus (HIV)	HIV-1 infection	2003	(48)
Enfuvirtide	Fuzeon^®^	Synthetic	Polypeptide	Membrane fusion inhibitor	Subcutaneous	Human immunodefiency virus (HIV)	HIV-1 infection	2003	(13, 15, 19, 49)
**Antifungal drug**									
Anidulafungin	Eraxis^®^	Semisynthetic derived from a fermentation products of *Aspergillus nidulans*	Cyclic lipopeptide	Inhibitor of the beta-(1,3)-D-glucan synthase	Intravenous	Candidemia and Candida infections	Antifungal drug	2006	(48)
Caspofungin	Cancidas^®^	Semisynthetic derived from a fermentation product of the fungus *Glarea lozoyensis*	Cyclic lipopeptide	Inhibitor of the beta-(1,3)-D-glucan synthase	Intravenous	*Candida* sp and *Aspergillus* sp	Antifungal drug	2001	(48)

*N.D., no data*.

#### Antiviral Activities

Some AMPs may present their activities against viruses. Antiviral peptides (AVPs) can cause membrane instability by integrating into viral envelopes. Both enveloped RNA and DNA viruses can be targeted by AVPs. AVPs can (i) interact with different glycoaminoglycans present on the cell surface competing with the virus for cellular binding sites, (ii) block the viral entry into the cell, (iii) suppress the cell fusion by interfering with the activity of ATPase protein, (iv) suppress viral gene expression, or (v) interfere with the assembly process of the viruses (13, 18, 19). As examples among the AVPs approved by the FDA, the polypeptide *enfuvirtide* that is a membrane fusion inhibitor block virus (HIV-1) from entering the host cells and *azatanavir*, an inhibitor of HIV-1 proteases, preventing the maturation of the proteins needed to assemble the viral capsid, can be cited (Table 1).

#### Antifungal Activities

Some AMPs pass through the fungal membrane by pore formation or act on beta-glucan or chitin synthesis and others interact with the membrane and cause cell lysis of fungi. They can lead to fungi death by (i) the inhibition of DNA, RNA, or protein synthesis, (ii) induction of apoptosis, (iii) permeabilization of membrane, and (iv) inhibition of cell wall synthesis and enzyme activity (18). Thus, the cyclic lipopeptides (*anidulafungin* and *caspofungin*) that are used as antifungal drugs are the inhibitors of the beta-(1,3)-D-glucan synthase (Table 1).

#### Immunomodulatory Activities

In addition to a broad spectrum of antimicrobial activities, AMPs have anti-inflammatory and immunomodulatory properties. They are described as the effective modulators of inflammation and neutralizers of toxins. AMPs can indirectly promote pathogen clearance of the host by stimulating chemotaxis (by recruiting/activating immunocytes), immune cell differentiation, and the initiation of adaptive immunity, while also preventing harmful inflammation and sepsis by the inhibition of cytokine release and direct scavenging of bacterial endotoxins (20, 50). Indeed, some AMPs modulate host immunity by influencing Toll-like receptor (TLR) recognition of microbial products (i.e., the neutralization of bacterial products such as lipopolysaccharide and lipoteichoic acid to suppress inflammation) and nucleic acids released upon tissue damage to promote auto-inflammation (51) (Figure 1). AMPs having immunomodulatory activities belong to the two major families: defensins and cathelicidins. Human defensins exhibit chemotactic properties and are produced by multiple cell types (i.e., neutrophils, macrophages, lymphocytes, and intestinal epithelial cells). The human cathelicidin membrane disrupting peptide *LL-37* acts as a chemoattractant for monocytes, neutrophils, mast cells, and T cells (11, 49, 51). In addition, the immunomodulatory properties of several marine-derived AMPs have been demonstrated. Thus, defensins from oyster or mytilus are the AMPs acting as host defense peptides that disrupt the membrane of microbial pathogens and play a major role in immunomodulation by acting on the innate and adaptive immune response (29). Among the approved AMPs that are used as antibacterial agents, the glycopeptide *vancomycin* exhibits pharmacological activity against Gram-positive bacteria and immunomodulatory activity affecting tumor necrosis factor-alpha (TNF-α) pathways. Thus, Arbabanel et al. showed that oral vancomycin can be used as an effective treatment for concomitant primary sclerosing cholangitis and inflammatory bowel disease in pediatric. Indeed, oral vancomycin-mediated disease resolution is associated with elevated peripheral TGF-β levels without alterations in Th1 or Th2 cytokine production patterns and increased regulatory T-cell levels (52).

### Advantages and Limitations of AMPs

**Figure 2:**
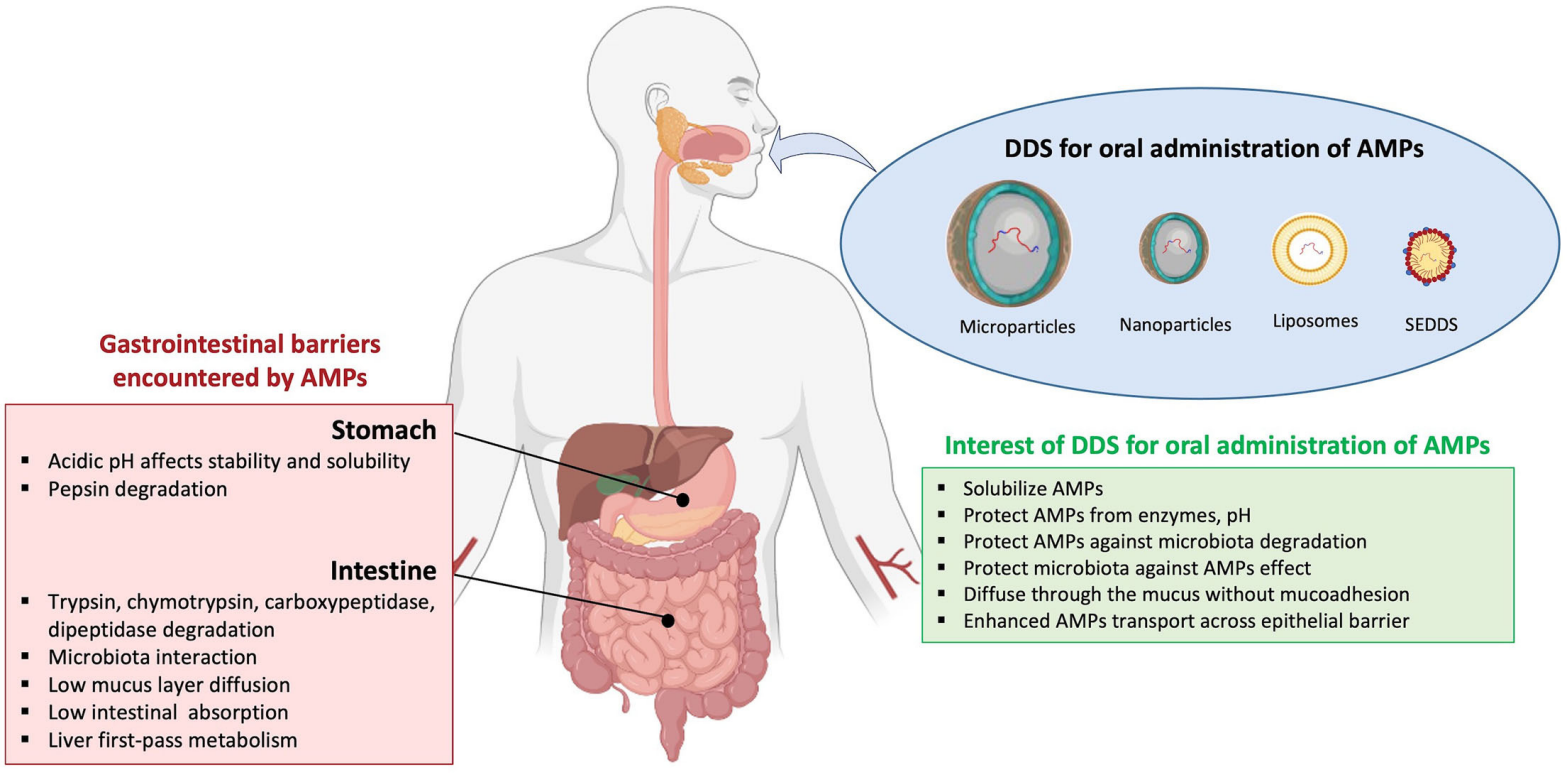
Barriers of AMP absorption and interest of drug delivery systems by oral route. Current drug delivery systems (DDS), including microparticles, nanoparticles, liposomes, and self-emulsifying drug delivery systems (SEDDS), are assessed for oral antimicrobial peptides (AMPs) administration. The encapsulation of AMPs in DDS presents advantages to avoid gastrointestinal barriers.

Antimicrobial peptides have a broad spectrum of antimicrobial activities (antibacterial, antiviral, and antifungal) and are a promising class of drugs to face the development of MDR pathogens. They have advantages over conventional antibiotics or antifungals, which include slower emergence of resistance, antibiofilm activity, and an ability to modulate the host immune response. AMPs are less immunogenic than recombinant proteins and antibodies. In addition, they are in general considered to have a safety profile because their metabolites are natural amino acids and there are having short half-life, few peptides accumulate in tissues (10, 13). However, despite the beneficial properties of AMPs, they present some limitations such as: (i) short half-life because of a rapid degradation by proteolytic enzymes, both in the bloodstream and in the gastrointestinal system; (ii) plasma protein binding, which leads to their inactivation; (iii) low metabolic stability and low oral bioavailability; (iv) rapid excretion through the kidneys and liver; (v) high toxicity (i.e., nephrotoxicity) and immunogenicity; (vi) a poor correlation between *in vitro* antimicrobial activity and their efficacy *in vivo*; and (vii) high costs of production (22, 42, 50) (Figure 1). For these reasons, their use for *in vivo* applications has not been fully satisfactory and to date, only a few AMPs are approved by the FDA for clinical use (Table 1). Most of the commercialized AMPs (except colistin and polymyxin B) are used for treating Gram-positive bacterial infections, and the development of AMPs to treat Gram-negative bacterial infections is needed. Moreover, concerning the route of administration, therapeutic peptides are mostly restricted to topical administration (skin and eye infections) or to parenteral administration, while the oral route (p.o) is mostly convenient for medication adherence. Indeed, p.o provides treatment acceptability for patients and facilitates the administration with non-invasive and ambulatory treatments; it is also the main route for local treatment of the gastrointestinal tract. Accordingly, it constitutes the *first* investigated administration route during the pharmaceutical development of a new drug. Unfortunately, this oral administration route is most of the time not suitable for the emerging therapeutic peptides, and significant efforts to knock down the locks of peptide administration by p.o are conducted. Indeed, after oral administration, peptides are exposed to the aggressive biological environment (pH, enzymes, and gut microbiota) and a complex structure (mucus, epithelial barrier, and hepatic *first* pass) of the gastrointestinal tract before they have local action or reach the systemic circulation (Figure 2). Generally, gastrointestinal degradation and low permeability lead to oral peptide bioavailability <1–2% (53).

### Indication of AMPs After the Oral Administration of Conventional Dosage Forms

#### Local Treatment of Gastrointestinal Diseases

Some peptides are already used or currently used in clinical trials to treat local diseases of the gastrointestinal tract, including colon infection disease such as amoebiosis and *C. difficile* or Enterococcus infections. First, some AMPs present an interest after oral administration without pharmaceutical forms. For example, Stiefel et al. (54) administered ramoplanin, a glycolipodepsipeptide, in drinking water (at 100 μg/ml of water) in female mice and demonstrated that it could potentially be used to reduce cross-transmission of vancomycin-resistant Enterococcus. Nevertheless, this work demonstrated some limitations notably due to the administration of this AMP in drinking water that, for example, may not reproduce the pharmacokinetic of human dosing.

LFF571, a new investigational thiopeptide, was synthesized to treat *C. difficile* infections. The aqueous solubility of this analog was enhanced in comparison with 4-aminothiazolyl analogs of the antibiotic natural product GE2270 A. Pharmacokinetic studies of the infected hamster were performed, and LFF571 solution (prepared with PEG-400 or Cremophor^®^EL, two solubilizing agents) was administrated at a dosage of 20 mg.kg^−1^ (55). A very low bioavailability was observed (as expecting to treat local *C. difficile* infection) and no detectable levels of *C. difficile* toxins A and B were measured in cecal content (animals were successfully treated). A randomized clinical study (phase II exploratory study in USA and Canada) was performed to compare LFF571 and vancomycin safety and efficacy (56). Moreover, a study was conducted to evaluate the safety, tolerability, and pharmacokinetic of LFF571 in healthy human volunteers (57). In both studies, LFF571 was well-tolerated in patients and in comparison with vancomycin administration, and the frequencies of serious adverse events were similar. Unfortunately, this phase II study was discontinued in 2019.

Moreover, to date, a few small peptides are on the market: peptides with good stability and high potency. These peptides are administrated with conventional forms [composed of active pharmaceutical ingredient (AMPs) and excipient(s)]. They include solid dosage forms such as tablets, capsules, powders, granules, lozenges, and liquid dosage forms such as solutions, emulsions, suspensions, and syrups. For example, vancomycin is administered by the oral route for both local and systemic delivery (Table 1). For local delivery, vancomycin is formulated in 125- and 250-mg capsules or in powder for oral solution to treat the infection of the intestinal mucosa (pseudomembranous colitis induced by *C. difficile*). Indeed, after oral administration, vancomycin is not usually absorbed into the blood and is excreted almost exclusively in the feces. However, absorption is reported in patients with inflammatory disorders of the intestinal mucosa (47). Nisin containing pectin/hydroxypropyl methyl cellulose (HPMC) compression-coated tablets was formulated for the treatment of local colon diseases such as irritable bowel syndrome, inflammatory bowel disease, and ulcerative colitis (58). Authors propose to coat a tablet with pectin and HPMC (two hydrophilic polymers, which swell to form a hydrogel layer upon contact with aqueous media) to form an enzymatically controlled delivery system. Adding pectin limits the hydration and swelling of HPMC that allow to maintain tablet integrity and thus nisin stability. Furthermore, *in vivo* studies with pectin/HPMC-coated tablet formulation conducted in healthy volunteers have demonstrated an interesting interplay between the tablet position in the gut, the hydration of the matrix, and the subsequent release pattern. Thus, HPMC maintains tablet integrity until the colon (59). For surotomycin (CB-315), peptides were proposed to treat diarrhea with severe *C. difficile* infection, tablet, and age-appropriate oral solid formulation that can be dispersed or dissolved. During clinical trials phase I, an insignificant absorption of surotomycin was observed and the primary route of elimination was in the feces following oral administration (60). Throat lozenge pharmaceutical forms were also developed to administer tyrothricin by the oral route in the treatment of patients with acute pharyngitis. Finally, liquid forms prepared from powders were used. Indeed, teicoplanin, to treat diarrhea with severe *C. difficile* infection, is presented as powder for oral solution at the dosages of 100, 200, and 400 mg. During pharmacokinetic studies, when teicoplanin is administered by the oral route at a 250- or 500-mg single dosage to healthy subjects, it was demonstrated that teicoplanin was not detected in the serum or urine but only recovered in feces (about 45% of the administered dosage) as unchanged medicinal product (44). Colistin sulfate was administered orally as a suspension *via* a nasogastric tube in the gastrointestinal tract for selective digestive tract decontamination in intensive care units (43).

#### Systemic Treatment After the Oral Administration of AMPs

Despite limited oral absorption of peptides, some of them are still administered orally and indicated for systemic bacterial or viral infections. For example, capsules (200 mg) of a HIV protease inhibitor, atazanavir (ATV), are on the market. ATV has a low oral bioavailability, which can be enhanced clinically by a co-administration with ritonavir and food (Table 2). To avoid food and ritonavir co-administration uses, amorphous solid dispersion system of ATV was also prepared with sodium lauryl sulfate as a carrier and polyoxylglycerides (Gelucire^®^ 50/13) as an absorption enhancer (Table 2). ATV solid dispersion showed a 4.7-fold increase in bioavailability compared with ATV alone (61).

**Table 2:** Oral AMP formulations to improve bioavailability.

**AMP**	**Formulation** **(trade name)**	**Interest of the formulation**	**Oral bioavailability of drug alone**	**Increased oral bioavailability with formulation/AUC0-∞/AUC0–t (μg/mL)**	**Relative bioavailability**	**References**
Azatanavir	Co-administered with ritonavir and food	- Inhibit cytrochrome P450 3A - Inhibit P-gp	19.6%	AUC_0−∞_= 3.58 μg. h. mL^−1^	100%	(61)
	Solid dispersion using sodium lauryl sulfate and Gelucir^®^ 50/13	- Improve dissolution rate and amount (amorphous state) - Absorption enhancer		AUC_0−∞_= 1.56 to 3.33 μg. h. mL^−1^	43–93%	(61)
	SNEDDS	- Enhancement of oral bioavailability of lipophilic drug - Bypass hepatic portal route - Promote the lymphatic transport of lipophilic drugs		AUC_0−12_ ≈ 1 μg. mL^−1^	N.D.	(62)
	Eudragit^®^ RL 100 nanoparticles	- Improve intestinal permeability (2.11-fold)	Low AUC_0−24_	AUC_0−24_ = 1.407 ± 2.18 μg. mL^−1^ at 8.01 h	N.D	(63)
Daptomycin	Proliposome	- Protect against harsh conditions presented in the GI tract - Improve oral absorption	low	AUC_0−*t*_ 46.39 ± 5.69 μg.h. mL^−1^	N.D.	(64)
Polymyxin B	Alginate microparticles	- Protect to gastric environment - Absorbed by a lymphoid transport across M cell in the follicle-associated epithelium - Prolonged serum levels compared with the drug dosed in a water solution	N.D (serum level peak ≅ 0.2 mg mL^−1^ at 48 h)	/	/	(65–67)
	Niosome	- Improve stability in simulated gastrointestinal fluids - Absorbed through M-cells	N.D.	AUC_0−48*h*_ 0.398 ± 0.03 mg. h. L^−1^	N.D.	(68)
Vancomycin	Water-in-oil-in-water multiple emulsion		<2%	Between 1.2 and 2.3 μg. h. mL^−1^	Between 30 and 50%	(69)
	Microemulsion	- Micelle formation - Pgp inhibition		AUC_0−6*h*_ between 12.94 ± 1.26 to 39.17 ± 6.30 μg. h. mL^−1^	N.D	(70)
	SEDDS	- Improve mucus permeation - Improve intestinal permeation (4–8-fold compared to free vancomycin solution)		N.D	N.D	(71)
	Folic acid-coated liposome	- Improve intestinal permeability	1.74%	AUC_0−last_ = 1.40 mL^−1^.min.kg^−1^	21.8%	(72)
	Tetraether lipid liposomes	- High stability in gastrointestinal fluids	1.5%	N.D.	4.8% (after 1h)	(73)

*N.D., no data; AUC, Area under the curve; SNEDDS, Self-nanoemulsifying drug delivery system; SEDDS, Self-emulsifying drug delivery system; GI, Gastrointestinal*.

Currently, vancomycin was administered by the oral route only for local *C. difficile* treatment. Indeed, after oral administration, during pharmacokinetic studies in human adults and during multiple dosing of vancomycin hydrochloride capsule at 250 mg every 8 h for seven dosages, no blood concentration was detected (74). So, to extend the use of vancomycin, pharmaceutical forms have been studied to improve its oral bioavailability. For example, multiple emulsion (water-in-oil-in-water) was proposed to administer vancomycin (as the model drug). The emulsion incorporating C18 unsaturated fatty acid oil (linoleic acid or linolenic acid) has improved the bioavailability more than 40% after emulsion administration into the rat colon loop (at a dosage of 5 mg.kg^–1^ of vancomycin) (69) (Table 2). On the other hand, for emulsion incorporating C18 unsaturated fatty acid oil (oleic acid) or docosahexaenoic acid, enteral bioavailability was lower (more than 25%) (69). Similarly, the formulation of a solution with a co-administration of the surfactant as an absorption promoter [PEG-8 caprylic/capric glycerides (Labrasol^®^) and D-α-tocopheryl polyethylene glycol 1,000 succinate (TPGS)] was studied (70). Vancomycin solutions were administered at a dosage of 20 mg.kg^–1^ into the rat ileum. Formulation containing 50% of Labrasol^®^ and 12.5% of TPGS increased the AUC_0−6h_ value of the vancomycin about 2.4 times in comparison with the formulation with 50% of Labrasol^®^ without TPGS (70).

Thus, the excipients that are used in pharmaceutical forms have demonstrated their importance to improve the absorption of AMPs. In addition, the stability, biocompatibility, safety, and efficacy of AMPs can be further improved through novel formulation strategies and design of new DDSs.

## DDSs for the Oral Administration of AMPs

After oral ingestion peptides can be easily affected by an biological environment; pepsin, protease (trypsin and chymotrypsin), acidic, or neutral pH, temperature resulting in the loss of their bioactivity in the gastrointestinal tract. Moreover, after oral administration, to obtain systemic delivery, besides potential gastrointestinal degradation, peptides have also to be transport across mucosal, epithelial, and endothelial barriers (75). Broadly, different approaches to update the inconvenience of conventional dosage forms were proposed to protect and/or to improve intestinal absorption of peptides. In this context, a number of approaches such as chemical modification of the peptide structure, a co-administration of absorption enhancer and/or protease inhibitor and/or mucolytic agents, or peptide encapsulation in well-adapted DDSs are proposed (76, 77).

Drug delivery systems exhibit different chemical or physical properties; they can be a protected drug, modified drug pharmacokinetic, controlled drug release, and improved therapeutic efficacy with less side effects of drugs (78). Thus, these systems emerge as pioneering and promising forms to enhance therapeutic effectiveness. DDSs are particulate pharmaceutical forms such as microparticles and nanoparticles (including liposomes, polymer- and lipid-based nanoparticles, and micelles) that allow drug encapsulation (Figure 2).

### DDSs for Gastrointestinal Diseases

The loading of AMPs into DDS could protect peptides from enzymatic degradation after oral administration for local treatment of the gastrointestinal tract. For example, the loading of nisin into the DDS is interesting due to its loss of bioactivity after an interaction with food (inactivation by enzymatic degradation, inactivity at alkaline pH). To demonstrate the interest of encapsulation, different nanoparticles as well as microparticle system were developed (79–85), but even if encapsulation improves the stability of nisin at alkaline pH or in the presence of enzymes, no pharmaceutical indications were described after oral administration. Thus, these different works demonstrated the interest of DDS to protect the peptide from gastrointestinal media. Similarly, the encapsulation of microcin J25 (the peptide with a bactericidal activity against a range of pathogenic enteric bacteria such as Escherichia coli and Salmonella) into the liposome coated with whey protein and pectin has protected significantly the peptide during *in vitro* digestion study (86). Finally, the efficacy of orally administered encapsulated cryptdin-2 onto chitosan nanoparticles (more than 105 nm particle size) was also demonstrated against Salmonella Typhimurium infection in mice (87). The property of chitosan can modulate the intestinal behavior of nanoparticles, which increase stability and protect cryptdin-2 against gastrointestinal conditions.

### DDSs for Systemic Delivery

To enhance the absorption of AMPs following oral administration, microparticulate and more specifically, nanoparticulate systems introduced advanced features. Indeed, due to their size, surface charge, and/or targeting moieties on the surface, nanoparticles are expected to diffuse through the mucus layer and to transport peptides across the intestinal barrier to reach blood circulation by different pathways (mainly transcellular pathways) (88). As a consequence, a lot of nanoparticulate delivery systems are described in the literature to improve the oral bioavailability of AMPs (Table 2; Figure 2).

#### Self-Emulsifying DDSs

Self-emulsifying DDS (SEDDS) and self-nanoemulsifying DDS (SNEDDS) are defined as the isotropic mixtures of oil, surfactants, and co-solvents. They present advantages for oral drug delivery like the protection against enzymatic degradation, reduced *first* pass metabolism, exhibiting mucus permeating properties, and enhanced absorption (89). Accordingly, daptomycin was incorporated into SEDDS that was prepared with tricaprylin (Dermofeel^®^MCT) and mono-diglyceride of medium chain fatty acids (mainly caprylic and capric) (Capmul^®^ MCM) as oil and PEG-40 hydrogenated castor oil (Cremophor^®^ 40) as the surfactant. Daptomycin was studied for the treatment of complicated skin infections, bacteremia, and right-side endocarditis caused by multiresistant Gram-positive bacteria. *In vitro* mucus permeation study demonstrated that SEDDS formulation improved daptomycin permeation by a factor 2 in comparison to pure daptomycin and protected daptomycin against enzymatic degradation (90). Similarly, SEDDS containing vancomycin, 25% of glycerol monocaprylate (Capmul^®^ 808G), 37.5% of PEG-40 (Cremophor^®^ R40), 13.6% of diethylene glycol monoethyl ether (Transcutol^®^ HP), and 26.5% of dimethyl sulfoxide (DMSO) was developed. SEDDS formulation improved intestinal *in vitro* mucosa permeating properties, nearly 45% of vancomycin permeated the mucus in SEDDS formulation within 4 h, whereas <5% permeated with free vancomycin (Table 2). *Ex vivo* permeation across porcine small intestinal mucosa confirmed these results (30 vs. 5% with SEDDS formulation and free vancomycin, respectively) (71). SNEDDS formulation of ATV was also developed. Optimized formulation was prepared with glyceryl monolinoleate (Maisin™35-1) as oil and diethylene glycol monoethyl ether (Transcutol^®^P) as the surfactant. After oral administration into rats at a dosage of 7.2 mg.kg^–1^ of ATV, the area under the curve (AUC) of ATV was improved 2.57-fold with SNEDDS compared to pure ATV (administered in the form of 0.3% carboxymethyl cellulose suspension) (62) (Table 2).

#### Microparticle Delivery System

Microparticles (microspheres or microcapsules) are defined as particles with size larger than 1 μm. They are promising encapsulation systems for protecting peptides from degradation, enhancing peptide stability, and providing an increased surface to volume ratio for peptide release and gastrointestinal absorption (91). In this way, oral polymeric microparticulate as a carrier of polymyxin B was developed. Polymyxin B, which has a potent bactericidal activity against a broad range of Gram-negative bacteria (e.g., *Pseudomonas aeruginosa*), is not absorbed orally. After encapsulation into crosslinked alginate/chitosan microparticles, the biological activity of polymyxin B was conserved due to microparticle stability in a gastric environment (Table 2) (65–67). Moreover, these microparticles demonstrated their ability to target the gut-associated lymphoid tissue (GALT) by Peyer's patch uptake, but more experiments have to be performed to demonstrate an eventual improvement of polymyxin B absorption (67).

#### Liposome Delivery System

Liposomes (vesicles in which an aqueous volume is entirely surrounded by a bilayer phospholipid membrane) are the *first* nanoparticulate delivery systems that reached the market (92). Conventional formulations of liposomes were instable in the gastrointestinal environment, but the modification of their composition by incorporating a specific phospholipid (e.g., DSPC, DPPC, and tetraether lipids) (92, 93) or by polymer coating (e.g., chitosan and PEG) (93, 94) enhanced their stability. Recent research on oral delivery systems has shown that liposomes can be employed to improve the bioavailability of encapsulated materials by protecting them against the chemical or enzymatic degradation environment and by improving their intestinal absorption (95). Uhl et al. (73) prepared a vancomycin-loaded liposome containing glycerylcaldityltetraether lipid (TEL). For *in vivo* studies, different vancomycin formulations were administered by gavage in Wistar rats. The obtained bioavailability 1 h after oral administration demonstrated that a 3-fold increase of vancomycin was observed by using TEL-liposome. Anderson et al. (72) formulated another vancomycin-loaded liposomes coated with folic acid to target intestinal epithelial cells expressing folic acid receptors. After the oral administration of vancomycin formulations (61.75 mg.kg^−1^) in Sprague–Dawley rats, the absolute bioavailability was 1.74, 6.7, and 21.8% from vancomycin solution, uncoated liposome, and folic acid-coated liposome, respectively. Thus, the folic acid and the liposomal formulation increase by a 12.5-fold the vancomycin bioavailability compared to vancomycin solution. In the same way, Arregui et al. (64) prepared a proliposome formulation [defined as dry, free-flowing particles coated with phospholipids, which can immediately form a liposomal suspension when in contact with water (96)] containing diacetyl phosphate and stearylamine to increase drug loading and to enhance the oral absorption of daptomycin. Pharmacokinetic studies in rats demonstrated that, after the oral administration of daptomycin at a dosage of 40 mg.kg^–1^, a greater AUC_0−t_ (46.39 ± 5.69 μg/ml/h), and higher C_max_ (8.35 ± 0.64 μg/ml) were observed with proliposomal compared to free drug (AUC_0−t_ and C_max_ were less than the limit of quantification) (Table 2).

#### Nanoparticle Delivery System

Nanoparticles (including nanospheres and nanocapsules) are lipid- and polymer-based nanocarriers. These nanoparticle approaches have some advantages for oral administration by protecting the encapsulated drug from enzymatic degradation, facilitating mucus diffusion, and membrane permeation (45). All these systems have already demonstrated their ability to improve peptide bioavailability (97–99).

Concerning AMPs, niosomes were developed. Niosomes (lipid-based nanoparticles) are similar to liposomes, but a bilayer is formed by a non-ionic surfactant and stabilized by the addition of cholesterol. These particles show a high stability in the gastric environment and a high permeability across the intestine (100). Polymyxin B niosomes were prepared using sorbitan monostearate (Span^®^ 60) and cholesterol. Vesicles were stable in simulated gastric and intestinal fluids, and about 86.2 and 78.5% of polymyxin B were retained, respectively (Table 2). Pharmacokinetic studies (at 2.0 mg.kg^–1^ dosage of polymyxin B in rats) demonstrated that polymyxin B niosome administrated orally present pharmacokinetic parameters (AUC_0−48_, *t*_1/2_) similar to polymyxin B sulfate administrated intravenously (68).

Polymeric nanoparticles provide also the possibility to enhance oral absorption. Eudragit^®^ RL 100 [a copolymer of poly (ethylacrylate and methyl-metacrylate)] nanoparticles were prepared by nanoprecipitation to encapsulate *ATV*. During *in vivo* pharmacokinetic studies in rats (at 7.2 mg.kg^−1^ dosage of ATV), the values of *C*_max_ and AUC_0−24_ increased to 1.1- and 2.91-fold, with pure drug and optimized nanoparticle formulation, respectively. These results demonstrate the considerable performance of the nanoparticulate DDS in enhancing the bioavailability of ATV (63).

## Conclusion

To date, there have been numerous studies investigating the interest of AMPs. Indeed, they display an antibiotic alternative and have already demonstrated their great potential to treat infectious diseases, including those caused by MDR strains. To be approved as therapeutic agents, AMPs must overcome their disadvantages, which limit their administration. Thus, in terms of future perspectives, one of the biggest challenges will be the development of pharmaceutical forms of the AMPs for all routes of administration and especially, oral administration, the main convenient administration route. Nevertheless, formulated with conventional forms, AMPs are always subjected to their limitations (enzymatic degradation, low bioavailability, rapid metabolism, opsonization, etc.). By this way, nano- and micro-DDSs emerge to play a critical role in determining the success of current and future products. Indeed, nanotechnology is an emerging field that offers a unique potential in comparison with conventional forms, including, for example, the protection against biological environment, the transport through barrier and cells, the improvement of bioavailability, or release modification. In this review, we have described the significance and high potential of AMPs to treat both systemic and local gastrointestinal infectious diseases as well as the oral AMP delivery systems available as innovative formulations. These new systems have to overcome the lack of specific regulatory guidelines notably for their characterizations (the absence of harmonized standard protocols), the complexity of their process of formulation and scale-up, and the uncertainties of their toxicity. They also require additional *in vivo* pharmacokinetic studies. In future, the encapsulation of AMPs into the DDS and their use in synergy with conventional antimicrobial drugs could be promising to obtain more effective oral treatments especially to treat MDR pathogen infections.

## Author Contributions

ER conceived the original idea. MA initiated the literature research and contributed Figure 2. CD, VA-M, and ER organized the literature research and wrote the final version. All authors contributed to this article and approved the submitted version.

## Funding

MA was funded by NANOMED EMJMD supported by the European Union and the Erasmus+ Program by the European Union in the Framework Agreement Number 2016–2057/001-001 EMJMD, No. 574439-EPP-1-FR-EPPKA1-JMD-MOB.

## Conflict of Interest

The authors declare that the research was conducted in the absence of any commercial or financial relationships that could be construed as a potential conflict of interest.

## Publisher's Note

All claims expressed in this article are solely those of the authors and do not necessarily represent those of their affiliated organizations, or those of the publisher, the editors and the reviewers. Any product that may be evaluated in this article, or claim that may be made by its manufacturer, is not guaranteed or endorsed by the publisher.
